# Retraction: Rapamycin-loaded nanoparticles for inhibition of neointimal hyperplasia in experimental vein grafts

**DOI:** 10.1186/1749-8090-7-17

**Published:** 2012-03-06

**Authors:** Junjie Zou, Xiwei Zhang, Hongyu Yang, Yi Zhu, Hao Ma, Shui Wang

**Affiliations:** 1 Department of General Surgery, the First Affiliated Hospital of Nanjing Medical University, Nanjng, Jiangsu province, China; 2 Division of Vascular Surgery, Department of General Surgery, the First Affiliated Hospital of Nanjing Medical University, Nanjng, Jiangsu province, China

## Retraction

This article [Zou *et al.*, Journal of Cardiothoracic Surgery 2011, 6:69] has been retracted at the request of the Editor [1]. Although the authors withdrew their submission the article was subsequently transmitted to the journal's production department, which resulted in it being published in error.

## References

[B1] Zou J Zhang X Yang H Zhu Y Ma H Wang S Rapamycin-loaded nanoparticles for inhibition of neointimal hyperplasia in experimental vein grafts Journal of Cardiothoracic Surgery 2011 6 69 10.1186/1749-8090-6-69 21569412 PMC3115851

